# Supplementary material for: Cerebral cortical thinning in Parkinson’s disease depends on the age of onset
Source: PLoS One. 2023 Feb 21;18(2):e0281987. doi: 10.1371/journal.pone.0281987 (PMC9942965; doi:10.1371/journal.pone.0281987)
Supplement: S1 Table — (DOCX) [file pone.0281987.s002.docx]

| **MNI coordinates**  **(x, y, z)** | **Cortical area** | **Cluster size**  **(mm^2^)** | **Clusterwise p-value** |
| --- | --- | --- | --- |
| Disease duration | | | |
| 4.9, -21.4, 63.3 | Right paracentral | 8432.90 | 0.0001 |
| 6.6, -80.3, 24.0 | Right cuneus | 1653.93 | 0.0005 |
| 38.2, 8.4, 41.5 | Right caudal middle frontal | 1050.17 | 0.0207 |
| -12.6, -15.1, 64.9 | Left precentral | 7064.21 | 0.0001 |
| LEDD | | | |
| 17.5, -97.7, 6.0 | Right lateral occipital | 1788.14 | 0.0001 |
| 26.7, -53.0, 45.3 | Right superior parietal | 1314.48 | 0.0033 |
| 23.1, -9.8, 52.5 | Right precentral | 1193.74 | 0.0087 |
| -28.2, -82.8, 15.7 | Left lateral occipital | 2880.21 | 0.0001 |
| -15.6, -15.2, 61.9 | Left precentral | 1723.14 | 0.0001 |

**Supplementary Table 1. Information on the identified clusters in Figure 1.**

MNI, Montreal Neurological Institute.
