# Supplementary material for: Cerebral cortical thinning in Parkinson’s disease depends on the age of onset
Source: PLoS One. 2023 Feb 21;18(2):e0281987. doi: 10.1371/journal.pone.0281987 (PMC9942965; doi:10.1371/journal.pone.0281987)
Supplement: S2 Table — (DOCX) [file pone.0281987.s003.docx]

**Supplementary Table 2. Information on the identified clusters in Figure 2(a).**

| **MNI coordinates**  **(x, y, z)** | **Cortical area** | **Cluster size**  **(mm^2^)** | **Clusterwise p-value** |
| --- | --- | --- | --- |
| LOPD | | | |
| 34.1, -42.5, 59.4 | Right superior parietal | 3949.14 | 0.0001 |
| 20.3, -59.1, 44.1 | Right superior parietal | 1460.23 | 0.0007 |
| 47.4, -73.7, 11.7 | Right lateral occipital | 1259.14 | 0.0024 |
| -27.2, -61.4, 28.4 | Left superior parietal | 3358.92 | 0.0001 |
| -54.5, 1.6, -7.0 | Left superior temporal | 1790.88 | 0.0001 |
| -5.9, -31.3, 56.1 | Left paracentral | 1485.73 | 0.0009 |

MNI, Montreal Neurological Institute; LOPD, late-onset Parkinson’s disease.
