# Supplementary material for: Cerebral cortical thinning in Parkinson’s disease depends on the age of onset
Source: PLoS One. 2023 Feb 21;18(2):e0281987. doi: 10.1371/journal.pone.0281987 (PMC9942965; doi:10.1371/journal.pone.0281987)
Supplement: S3 Table — (DOCX) [file pone.0281987.s004.docx]

**Supplementary Table 3. Information on the identified clusters in Figure2(b).**

| **MNI coordinates**  **(x, y, z)** | **Cortical area** | **Cluster size**  **(mm^2^)** | **Clusterwise p-value** |
| --- | --- | --- | --- |
| Disease duration | | | |
| -16.6, -70.2, 37.7 | Right superior parietal | 1630.79 | 0.0007 |
| LEDD | | | |
| 23.1, 57.5, 8.0 | Right rostral middle frontal | 1508.58 | 0.0005 |
| 59.5, -4.1, -1.1 | Right superior temporal | 1220.40 | 0.0032 |
| 31.0, -86.2, 3.4 | Right lateral occipital | 1123.85 | 0.0063 |
| 6.9, -34.9, 56.8 | Right paracentral | 953.76 | 0.0219 |
| -14.7, -65.8, 36.2 | Left precuneus | 2512.18 | 0.0001 |
| -45.7, -20.2, -2.2 | Left superior temporal | 1411.55 | 0.0013 |
| -63.0, -11.3, 16.4 | Left postcentral | 1240.06 | 0.0037 |
| -5.2, -35.5, 57.2 | Left paracentral | 1151.38 | 0.0054 |

MNI, Montreal Neurological Institute; LEDD, levodopa equivalent daily dose.
