# Supplementary material for: Cerebral cortical thinning in Parkinson’s disease depends on the age of onset
Source: PLoS One. 2023 Feb 21;18(2):e0281987. doi: 10.1371/journal.pone.0281987 (PMC9942965; doi:10.1371/journal.pone.0281987)
Supplement: S4 Table — (DOCX) [file pone.0281987.s005.docx]

**Supplementary Table 4. Information on the identified clusters in Figure 3.**

| **MNI coordinates**  **(x, y, z)** | **Cortical area** | **Cluster size**  **(mm^2^)** | **Clusterwise p-value** |
| --- | --- | --- | --- |
| E-MOPD | | | |
| 4.5, -22.3, 63.7 | Right paracentral | 3346.77 | 0.0001 |
| -36.9, -6.0, 53.0 | Left precentral | 2828.22 | 0.0001 |
| LOPD | | | |
| 37.0, -12.6, 60.2 | Right precentral | 3024.31 | 0.0001 |
| 45.0, -73.7, 11.6 | Right lateral occipital | 1236.40 | 0.0029 |
| 7.7, -80.7, 24.4 | Right cuneus | 858.83 | 0.0408 |
| -18.6, -63.0, 38.5 | Left superior parietal | 1604.00 | 0.0009 |
| -8.5, -19.4, 69.0 | Left precentral | 1125.50 | 0.0120 |
| -7.4, 29.1, 43.4 | Left superior frontal | 1066.99 | 0.0171 |
