# Supplementary material for: Cerebral cortical thinning in Parkinson’s disease depends on the age of onset
Source: PLoS One. 2023 Feb 21;18(2):e0281987. doi: 10.1371/journal.pone.0281987 (PMC9942965; doi:10.1371/journal.pone.0281987)
Supplement: Attachment — Submitted filename: Response_letter.doc [file pone.0281987.s006.doc]

December, 22 2022

Niels Bergsland

Academic Editor

*PLOS ONE*

Dear Editor:

Subject: Submission of revised paper “Cerebral cortical thinning in Parkinson's disease depends on the age of onset” (PONE-D-21-33205)

Thank you for your email enclosing the reviewer’s comments. I also would like to thank you for accepting my request to extend the deadline for submitting the manuscript. We have carefully reviewed the comments and have revised the manuscript accordingly. Our responses are given in a point-by-point manner below, and changes to the manuscript are shown in red.

**Response to Editor’s comments.**

*For example, was the enrollment period really nearly 20 years long? If so, it seems likely that there would have been major scanner changes throughout the course of the study.* *If so, it seems likely that there would have been major scanner changes throughout the course of the study. It is not clear how this would have affected the results. In addition, it is not clear what was done for patients with multiple scans, since the work presented seems to be cross-sectional in nature.*

Thank you for your comment. There was no change in the MRI model used for imaging during the observation period. In addition, given that we used images taken from the voxel-based specific regional analysis system for Alzheimer’s disease {Hirata, 2005 #323}, which is a dementia diagnosis software, there was no change in the imaging conditions.

As you said, this study is basically a cross-sectional study. Therefore, we believe that the effect is as large as the effect of the difference in the number of observations in longitudinal studies. However, it is desirable for all patients to have the same imaging frequency, and there is no certainty that there was no effect. This information is listed in the Discussion section.

**Response to Reviewer 1’s comment.**

*1. This appears to be a retrospective study, have the authors any intention of conducting a prospective one more aligned with their hypotheses?. How did the authors arrive at the study size of 62? It was not clear from the text.*

Thank you for your comment. We are currently conducting a four-year follow-up study of patients with Parkinson’s disease. In this ongoing study, the number of MRI scans of patients and the observation items for motor and non-motor findings are uniform. On the basis of the results of this study, we will also analyze the progression rate of brain atrophy associated with the age of onset.

This is because imaging was performed under the MRI imaging conditions specified in this study, and there were 62 patients who did not meet the exclusion conditions described in the Subjects section. Given that there was no description that the MRI imaging conditions were met, this information was listed in the Subjects section.

*2. It is not clear why the authors divided the PD group in younger and older. Why authors have opted to 63 years and not 40 years, 55 years?*

Thank you for your inquiry. The standard age of 63 years was based on the average age of onset of Parkinson’s disease in Japanese patients.

*3. An important concern is about the fact that the authors did not compare PD groups to age- and sex-matched healthy controls.*

*4. The specificity of these imaging results in terms of clinical meaning or physiopathology of PD is unclear: age or other factors may explain the results.*

Thank you for your comment. Given that the horizontal axis of the correlation analysis is not based on age but clinical years, a comparison with healthy subject data is not possible. However, considering that patients with a longer clinical course tend to be older, it is thought that aging has some effect on PD.

Regarding the effect of age, we incorporated age as a covariate during the image analysis process. Therefore, we believe that the effect has disappeared.

*5. There is little mention of the existing extensive literature about the changes and progression of expression of the morphological and functional changes in the brain related to the age of onset in PD with both MRI and PET.*

Thank you for this comment, and we apologize for this deficiency. However, no study has investigated the relationship between the age at onset of Parkinson’s disease and imaging tests such as MRI and PET.

**Response to Reviewer 2’s comment.**

*1. Appendix tables arent properly labeled.*

Thank you for pointing out this issue. We have labeled the tables in the appendix.

*2. The correlation presented with disease duration has no context as far as duration from diagnosed or personal notice.*

Thank you for this comment. The time of onset was defined as the time when patients became aware of motor symptoms. We have added this information in the Materials and Methods section.

*3. Were there any other clinical information, such as UPDRS or Hoehn-Yahr correlated with the significant differences? Otherwise the impact is limited.*

Thank you for your inquiry. Given that this is a retrospective study, we were unable to obtain the clinical information you indicated. We have indicated this information in the Discussion section. By using previous reports, we examined the relationship between the detected cortical atrophy sites and clinical symptoms.

We hope that the revised version is now suitable for publication, and we look forward to hearing from you.

Sincerely

Kazuhide Seo

Department of Neurology, Saitama Medical University, Saitama, Japan

38 Morohongo, Moroyama, Saitama 350-0495, Japan

Tel.: +81-49-276-1208

Fax: +81-49-295-8055

Email: seo.kazuhide@1972.saitama-med.ac.jp
